# Prenatal Genetic Testing for Beckwith‐Wiedemann Syndrome: Considerations, Challenges and Observations (A Real‐World Study)

**DOI:** 10.1002/pd.70206

**Published:** 2026-06-25

**Authors:** Melissa Connolly, Louise McClelland, Pierpaola Tannorella, Tanja Richter, Ester Mainini, Andreas Dufke, Annette Lischka, Matthias Begemann, Katja Eggermann, Thomas Eggermann, Silvia Russo

**Affiliations:** ^1^ West Midlands Genomics Laboratory Birmingham UK; ^2^ Research Laboratory of Medical Cytogenetics and Molecular Genetics IRCCS Istituto 2 Auxologico Italiano Milan Italy; ^3^ MVZ Genetikum GmbH Center for Human Genetics Neu‐Ulm Germany; ^4^ Institute for Medical Genetics and Applied Genomics University of Tübingen Tübingen Germany; ^5^ MVZ Genetikum GmbH Center for Human Genetics Stuttgart Germany; ^6^ Center for Human Genetics and Genome Medicine Medical Faculty RWTH Aachen University Aachen Germany; ^7^ Center for Genome Medicine Massachusetts General Hospital Boston Boston Massachusetts USA

## Abstract

**Objective:**

Prenatal genetic testing for imprinting disorders is rarely requested with the exception of Beckwith‐Wiedemann syndrome (BWS) which is associated with specific ultrasound findings (e.g., placental mesenchymal dysplasia, omphalocele). However, genetic testing for BWS is challenging as aberrant DNA methylation has to be addressed which often occur as mosaicism. As a growing number of requests for prenatal BWS testing is observed, data from genetic prenatal BWS testing was compiled to delineate its suitability as well as limitations.

**Method:**

Three European laboratories compiled the experiences from 646 prenatal samples. The samples were analysed by methylation‐specific assays targeting the imprinting centres in 11p15.5, in a sub‐cohort *CDKN1C* was sequenced.

**Results:**

The overall detection rate for BWS specific molecular disturbances was 9.75%, and the spectrum of alterations reflected that from postnatal BWS cohorts. The rate of failing samples was in total 4.3%, nearly all failures were observed in native samples.

**Conclusion:**

Prenatal testing for BWS should be considered in pregnancies with ultrasound findings or family history suggestive for BWS. In the majority of tests, evaluable results can be achieved, though mosaicism can never be excluded and therefore false‐negative results are possible.

## Summary

- What's already known about this topic?
  ◦ Beckwith‐Wiedemann syndrome is associated with several specific clinical features detectable by ultrasound.
  ◦ Prenatal genetic testing for BWS is challenging because of molecular heterogeneity and mosaicism.
- What does this study add?
  ◦ Based on a large number of prenatal cases from three experienced centres the reliability of prenatal genetic BWS testing is documented.
  ◦ Prenatal genetic testing for BWS should be considered in case of specific ultrasound findings or positive family history but should also as an additional prenatal option after NGS testing.

## 1 Introduction

Beckwith‐Wiedemann syndrome (BWS) belongs to the molecularly defined group of congenital imprinting disorders (ImpDis), which are characterised by common underlying (epi) genetic aetiologies (for review: [1]). ImpDis are associated with molecular changes in genes which are expressed from only one allele in a parent‐of‐origin specific pattern, that is genes regulated by genomic imprinting. Therefore, BWS like other ImpDis does not follow the classical modes of inheritance, but the sex of the parent contributing the affected alleles is relevant for the clinical features in the offspring. Molecular disturbances of ImpDis comprise changes in both genetic sequences (“genetic mutations”) and gene regulation (imprinting defects). Clinically, ImpDis share features within the same spectrum and manifest in early childhood, but both phenotypic and molecular disturbances may overlap [2]. Prenatal features of ImpDis are mainly nonspecific, with the exception of BWS (for review: [3]).

BWS is classified as an overgrowth disorder, characterized by a broad range of clinical features including asymmetry, organomegaly, macroglossia, abdominal wall defects, hypoglycemia, and an increased risk for embryonal tumours (for review: [4]). A scoring system for prenatal testing has been proposed by Carli et al. [5], based on the prenatal data from a large cohort of BWS patients and literature search. However, only a few symptoms are detectable during early pregnancy. Omphalocele, polyhydramnios, macrosomia and placental mesenchymal dysplasia or placentomegaly are typically observed by the 16th week of gestation; additionally there is an increased risk of preeclampsia [6].

At the molecular level, BWS is associated with alterations in the 11p15.5 region. More than 60% of the molecularly confirmed patients exhibit loss of methylation of the centromeric imprinting centre IC2 (*KCNQ1OT1*:TSS‐DMR; IC2 LOM), while nearly 20% carry a paternal uniparental disomy of 11p15.5 (upd(11)pat) [2]. Approximately 10% show gain of methylation at IC1 (*H19/IGF2*:IG‐DMR; IC1 GOM); and copy number variants (CNVs) only contribute a small proportion of cases. Finally, pathogenic sequence variants (SNVs) affecting the maternally expressed *CDKN1C* allele can be diagnosed in up to 5% of sporadic and in up to 50% of familial BWS cases.

Genetic testing for BWS is currently performed primarily by MS‐MLPA (methylation‐specific multiplex ligation‐dependent probe amplification), but other methods are also suitable provided they address methylation status [2, 4, 7, 8, 9]. However, diagnostic testing is challenging ‐because imprinting defects and upd(11)pat in BWS, often occur in a mosaic state. Current frequently utilised testing methodologies are often limited in their sensitivity for detection of low‐level mosaicism and therefore these imprinting defects may escape detection.

Prenatal testing for BWS is further complicated by factors related to gestation and cell lineage of the sample. Sample types available for testing consist of extraembryonal chorionic villi (CVS), whose (epi)genetic constitution may differ from the foetus, and foetal amniotic fluid (AF) (for review: [10]). The cell linages of the extraembryonal tissues and foetal cell mass have deviated from each other in embryogenesis and this can result in differences in distribution and levels of mosaicism between these different tissues. Furthermore, the time of sampling can influence results, as methylation is not established in all DMRs and CpGs in the placenta before the 13th week of gestation [11]. Accordingly, there is a risk of false positive or negative during prenatal methylation testing for BWS.

Due to these technical limitations and the absence of non‐specific nature of prenatal features for the majority of ImpDis, invasive prenatal testing is regularly only requested in case of a familial predisposition (CNV, SNV) or a chromosomal constitution prone to UPD formation [3]. With the increased application of ultrasound techniques and the implementation of array and next generation sequencing (NGS) based assays in the prenatal testing of pregnancies with pathological ultrasound results, our laboratories observe a growing number of requests for prenatal BWS testing, in order to comprehensively accomplish the diagnostic workup. In the following, we report our results from the largest cohort of prenatal samples referred for BWS testing reported so far.

## 2 Cohort

Diagnostic prenatal testing for 646 pregnancies in total was requested at the contribution centres (Aachen/AA: *n* = 197; Birmingham/BI *n* = 253; Milan/MI *n* = 196) (Table 1). The time of ascertainment included the years from 2017 to March 2025 in Aachen, 2008‐2024 in Birmingham, and 2006‐2025 in Milan. In UK BI is a specialist centre for BWS testing, in Italy MI is a reference centre for prenatal BWS testing, and in Germany AC is expert centre.

**TABLE 1:** Overview on the tissues and samples included in the study.

Samples		Native	Cultured	Unknown	Total
CVS	AA	29	12	23	64
	BI	39	5	0	44
	MI	63	26	11	100
	*Sum*	*131*	*43*	*34*	*208*
AF	AA	33	48	14	95
	BI	80	129	0	209
	MI	11	67	15	93
	*Sum*	*124*	*244*	*29*	*397*
Unknown	AA				38
	MI				3
	*Sum*				*41*

Abbreviations: AA, Aachen; AF, amniotic fluid; BI, Birmingham; CVS, chorionic villus sampling; MI, Milan.

All families gave informed consent for genetic testing, the study was approved by the ethical committee of the Medical Faculty of the RWTH Aachen (EK303‐18).

Terminations of pregnancy and cases in which maternal contamination was observed were not included in the study. Prenatal diagnoses sent for chromosomal rearrangements involving the chromosome 11 and present in parents or disclosed in foetus were not considered as well.

DNA from chorionic villus sampling (CVS) or amniotic fluid (AF) was extracted from both native as well as cultured cells. In the majority of cases, DNA was isolated by the referring laboratories, therefore a standardisation of sample preparation and culturing could not be achieved.

As the three contributing centres are diagnostic laboratories, the reasons for referral were not systematically provided, and data about the course and outcome of the pregnancies were often not available.

### 2.1 Subcohort Aachen/Germany (AA)

In general, the reason for referral were abnormal ultrasound findings, but detailed information was provided only in 85 cases with omphalocele as the far most frequent finding (85.0%).

The 197 samples comprised 64 DNAs isolated from native or cultured CVS and 95 from native or cultured AF. In 38 samples, information about the tissue was not provided.

### 2.2 Subcohort Birmingham/United Kingdom (BI)

The reason for referral was provided in 207 of the 253 cases. The most common referral reason in these cases was omphalocele (69.2%), followed by omphalocele plus other scan findings found in 12.6%.

AF samples represented the majority of referrals at BI (*n* = 209), compared to CVS (*n* = 44).

### 2.3 Subcohort Milan/Italy (MI)

The reason for referral was known in 178 out of 196 cases. Omphalocele (*n* = 141/196%, 71%,9%) was the most common clinical indication, including 17 cases with other malformations in the cohort with omphalocele (12.0%). The prenatal samples analysed in Milan were 47.4% AF (*n* = 93) and 51.0% CVS (*n* = 100); additionally, one foetal blood sample was examined and in two cases the tissue was unknown.

## 3 Methods

The majority of samples were analysed by semiquantitative MS‐MLPA using successive versions of the ME030 assay (MRC Holland, Amsterdam, NL), the diagnostic procedure corresponded to the manufacturer's protocol. In each run, at least three DNA samples extracted from peripheral blood from healthy donors (AA, BI) or from native CVS and AF samples (MI) were used. Due to the heterogeneity of referring labs, a common protocol for DNA extraction could not be used.

MS‐MLPA data in Aachen and in Milan were processed by the Coffalyser software (https://www.mrcholland.com/technology/software/coffalyser‐net).

In subcohort AA the quality of data was classified as acceptable in case the methylations‐specific probes were within the default arbitrary borders (thresholds of 0.7 for loss and 1.3 for gain) provided by Coffalyser.Net.

In subcohort MI, methylation values of samples were considered hyper or hypomethylated when they fell over/below +/−3SDS of methylation values in each probe obtained in a cohort of 50 healthy controls. Values between 1SDS and 2SDS were considered borderline values.

MS‐MLPA data in Birmingham was analysed using GeneMarker software (SoftGenetics). Intrasample and intersample comparison to 4 normal controls enables relative copy number and methylation status determination. Arbitrary borders (thresholds of 0.7 for loss and 1.25 for gain) were used to assess methylation status.

In the BI subcohort results were classified as “failed” when MS‐MLPA QC metrics were not passed, for example, low DNA input indicated by the DQ peaks, incomplete digestion.

Results were classified as “failed” in case the data did not fit the aforementioned borders.

In accordance with MRC Holland instructions all laboratories used the average methylation status of the probes within a DMR to determine the methylation status.

In Milan, 55 cases investigated before 2010 were tested using Southern blot approach for methylation and microsatellite segregation to establish uniparental disomy, as previously reported [12].

All samples were checked for maternal contamination.

Sanger sequencing of *CDKN1C* has been suggested as a second step of molecular BWS testing [4] and is therefore not conducted systematically but only if requested. Accordingly, exons and exon‐intron boundaries of the gene were analysed using standard Sanger sequencing methods only in a subset of the samples. Variant interpretation was primarily based on the ACMG criteria (American College of Medical Genetics, [13]? For BI cases, the ACGS variant interpretation best practice guidelines (Association for Clinical Genomic Science [14]) available at the time of analysis were used in conjunction with ACMG.

## 4 Results and Discussion

In total, 646 samples were referred for prenatal testing to the three labs. In general, it was confirmed that MS MLPA is a suitable tool for aberrant methylation testing in prenatal tissues. Though the outcome of the pregnancies could not be generally verified, we are aware of only one false negative result in a DNA sample isolated from prolonged amniotic cell culture (BI), the patient was confirmed to have IC2 LOM in a postnatal blood sample. In another case, an unexpected IC1 LoM (i.e. LoM of all probes targeting the DMR) was observed in a CVS suspicious for BWS, postnatally this case was confirmed to be a multilocus imprinting defect (MLID) with IC2 LOM (BI). There was no further feedback regarding possible false‐negatively tested pregnancies, we therefore assume that they are rare. However, their number cannot be precisely determined as information about the final outcome of these pregnancies was not provided.

As calculation controls, DNA samples from native CVS and AF samples were used as controls in one centre (MI), but in the other two contributing labs (AA, BI) it could be shown that samples isolated from native lymphocytes are suitable as well. The reason for choosing the latter is the fact that obtaining controls precisely fitting to the same sample type (tissue, gestational week (gw) and extraction method) in the prenatal setting can be difficult. Nearly all IC1 and IC2 probes in ME030 proved to be suitable for interpretation, with the exception of IC1 probe 301 nt (MRC Holland 06,266‐L05772) which only seemed to be reliable when matched tissues are used as controls.

### 4.1 Tissues and Methods

As generally observed for MS MLPA testing, the source and quality of DNA were the major causes of failure or reduced quality of results (Tables 2a and 2b). The rate of failing samples ranged from 2% to 9.4%, nearly all failures were observed in native samples (CVS and AF). However, it has to be considered that the contributing centres do not use a similar system to tier results as suitable and limited quality. The higher failure rate in CVS samples might be explained by failure of the MS‐MLPA digestion controls. It might be circumvented by the use of tissue matched controls, as illustrated by the date from the Milano group (Table 2b). However, there is evidence that DNA methylation is less stable in placenta and different from other tissues (e.g., [15]) and therefore might cause false results. Early gestation native AF samples with low yield DNA also contributes to the failure rate, but in general BWS testing of amniotic fluid samples proves to be robust, with no sample matching required.

**TABLE 2:** General testing results and numbers of failed samples. Overview on all samples (a) and details about the failed cases, documented in respect to tissue and culturing (b).

2a		
Result	Total	%
**Positive diagnoses** **^a^**	63	9.75%
**Normal**	555	85.91%
**Failed**	28	4.33%
***Sum***	*646*	*100.00%*

2b				
		Native	Cultured	Unknown
**CVS**	AA	0	0	1
	BI	13	0	0
**AF**	AA	3	0	0
	BI	8	3	0
	MI	0	0	0
***Sum***		*24*	*3*	*1*

Abbreviations: AA, Aachen; AF, amniotic fluid; BI, Birmingham; CVS, chorionic villus sampling; MI, Milan; for the definition of “failed” see text.

^a^ positive cases after methylation testing, *CDKN1C* results are not included but listed in Table 3b.

Based on our data from a large cohort of prenatal cases, prenatal diagnosis using native and late CVS has proven, in most cases, to be a reliable tissue for 11p15 methylation testing, despite the limitations discussed [4]. An advantage of CVS is the earlier sampling time, which allows clinical decisions regarding pregnancy management (continuation or termination; monitoring for preeclampsia, birth management (for review: [3]) to be made earlier in pregnancy. The timing of sampling must also be considered in the context of differential diagnosis for omphalocele, which is also a frequent finding in trisomy 18 and other inborn genetic constitutions, and is often detected on a first trimester ultrasound. In such cases, a patient may elect for a CVS.

No correlation was found between analysis success and week of gestation. However, two CVS samples from early pregnancies (< gw 14) referred because of omphalocele and placental mesenchymal dysplasia unexpectedly showed LOM at IC1. Postnatal samples were not available from these cases, but the birth of a healthy child was reported in the case of the mesenchymal dysplasia. It can be assumed that the methylation was not established at the time of sample drawing, or that the IC1 LOM was restricted to the placenta. In the second case the pregnancy was terminated because the severity of the omphalocele. In the autopsy report a growth delay was indicated. Therefore, MLID cannot be excluded in that case, but the aforementioned reasons might account for the molecular finding as well. We therefore suggest performing diagnostic testing on CVS no earlier than gw 13–14. Furthermore, it has to be considered that AF results better reflect the cell lineage of the foetus itself than CVS.

The alternative to CVS is an early gestational AF (e.g., gw 15–16). Earlier gestation AF samples will have low cellularity, resulting in poor quality uncultured DNA samples which is unsuitable for testing (Table 2b). Culturing these AFs is an option but it is important to monitor the length of time in cell culture, a longer time in culture can result in clonal features which do not correspond to foetal cell lineage, leading to a methylation result not reflective of the true methylation levels in the foetus. The low cellularity of earlier gestation AF samples often results in slow growing cultures and increasing the risk of false negative results. Hence when making decisions on prenatal sampling, the requirements of managing the pregnancy and the differential diagnosis for omphalocele must be balanced against the suitability of the prenatal sample for methylation testing. This highlights the importance to communicate the limitations of prenatal BWS testing to the clinicians and families.

In general, MS MLPA is a suitable tool for detecting aberrant methylation in prenatal tissues even when performing comparative analysis to DNA extracted from lymphocytes. In fact, also other techniques can be used for prenatal testing, and it has already been shown that their suitability is similar [12]. In any case, laboratories performing prenatal testing for BWS should have ample experience in testing for BWS postnatally and have experience in processing prenatal tissues or good links to a centre with this experience. The authors are aware of only one case of a false negative but acknowledge there is a lack of postnatal follow up data. Cases can be lost to follow‐up during transition to postnatal care, or due to lack of information provided to the testing laboratories to enable them to identify these postnatal samples are for follow up. Accordingly, collecting pregnancy outcome data to enable audit of false positive and negative rates is a major task for the future.

### 4.2 Diagnostic Yield

The diagnostic yield in the cohort averaged approximately 9.75% in all contributing centres (Table 3a and b). Although differences were observed between the laboratories diagnostic yield which likely reflect local testing eligibility criteria and national regulations. In fact, the diagnostic yield is lower than that of postnatal cohorts, but this observation can be explained by the non‐specificity of prenatal BWS features.

**TABLE 3:** Diagnostic yield in the three cohorts by methylation testing (a) and *CDKN1C* sequencing (b and c). (a) Molecular subgroups identified in our prenatal cohort and comparison of their frequencies with the literature.

3a									
Reference		Cohort size (*n*)	Positive cases^a^ (*n*)	Diagnostic yield^a^	Relative ratios of molecular subtypes among the solved cohorts				
					IC1 GOM	IC2 LOM	Upd(11)pat	MLID	Others
[2]	**Postnatal**	1272	150	24.9%	11.8%	64.0%	19.5%	12.8%	4.6%
(24)	**Prenatal**	166	NA^d^	NA	8.4%	59.6%	19.3%	NR	12.6%
								
(22)		122	20	16.3%	5.0%	75.0%	20%	NR	0
**This study**									
AA		197	13	6.6%	0	92.3%	0	7.7%	0.0%
BI		253	31	12.3%	3.2%	80.7%	6.5%	0.0%	IC1 LOM: 3.2% CNVs: 6.5%
MI		196	19	9.7%	10.5%	63.2%	10.5%^b^	10.5%	5.3% (upd(14)pat)^c^

3b	
	*CDKN1C* analysis
Cohort size (n)	136
Positive cases (n)	7
Diagnostic yield	5.1%
Inheritance:	
Maternal	4
De novo	2
Not determined	1

3c			
*CDKN1C* variant (NM_001122630.2)	ClinVar ID	Inheritance	Classification (based on ACMG criteria)
c.655 C > T, p.(Gln219Ter)	132,861	De novo	Pathogenic (PVS1, PM2_sup, PS4_mod)
c.655 C > T p.(Gln219Ter)	132,861	Maternal	Pathogenic (PVS1, PM2_sup, PS4_mod)
c.661 C > T p.(Gln221Ter)	210,647	Maternal	Pathogenic (PVS1, PM2_sup, PP5
c.786dup p.(Asp263ArgfsTer12)	NR	Maternal	Likely pathogenic (PVS1, PM2_sup)
c.812 C > G p.(Ser271Ter)	18,445	Maternal	Pathogenic (PVS1, PM2_sup, PP5)
c.845del p.(Ala282GlyfsTer28)	NR	Maternal	Pathogenic (PVS1, PM2_sup, PP5)
c.913 C > T p.(Arg305Trp)	1,406,444	de novo	Likely pathogenic (PP3, PM2_sup, PP5, PS2_mod)

Abbreviations: NA, not applicable; NR, not reported.

^a^ The *CDKN1C* testing results are not included but are listed in (b and c).

^b^ one of these cases was a paternal mosaic uniparental diploidy (“genomewide uniparental disomy”).

^c^ in one case upd (14)pat was identified). c Pathogenic variants identified in the cohort and their parental origin (ClinVar ID: variants already listed in ClinVar (https://www.ncbi.nlm.nih.gov/clinvar/), NR not yet reported, PCNA Proliferating Cell Nuclear Antigen region, NLS nucleolar localisation signal region, PS phosphorylation site, functional domain: in variants for which the domain is not listed it can be assumed that the have an impact on the structure of the variants, close to these variants other pathogenic variants have already been reported).

^d^ This study [21] summarised only positively tested cases.

In the majority of positively tested samples, IC2 LOM was observed. Although IC2 LOM is the most frequently reported molecular subtype, it represented a higher proportion of the diagnoses within this prenatal cohort than typically reported [4]. This overrepresentation might reflect the bias towards molecular subgroups associated with omphalocele (Table 4). Indeed, other signs such as macroglossia might be missed in case of early invasive prenatal testing as they become visible by ultrasound after the second trimester.

**TABLE 4:** Overview on the clinical findings and/or reason for referral in the positively diagnosed cases.

	IC1 GOM	IC2 LOM	Upd (11)pat	CDKN1C	Upd (14)pat	MLID
Number of cases	*N* = 2^a^	*N* = 43^a^	*N* = 2^a^	*N* = 3^a^	*N* = 1^a^	*N* = 1^a^
Omphalocele		35	2	7	1	1
Macroglossia	1	4				
Macrosomia	2	2				
Placental mesenchymal dysplasia		2	1			1
Organomegaly		4				Nephromegaly
Polyhydramnios	2	1				
Foetal malformations		3	1	2		
Bell‐shaped thorax						1

^a^ Number of cases per subgroup with clinical information.

Among the prenatal samples with IC2 LOM, three showed MLID, indicated by LOM of both imprinting centres in 11p15.5 targeted by the M030 MLPA assay (AA, MI). However, the precise number of MLID in our prenatal cohort is unknown because this constitution was not ascertained systematically by analysing further clinically relevant imprinted loci.

The upd (11)pat as the second frequent finding in postnatal BWS, might be underrepresented because omphalocele is a less common feature in carriers of this constitution. Among Milan cases mosaicism for a paternal uniparental disomy (“genomewide UPD”) in a foetus without omphalocele was identified, but mesenchymal dysplasia and different foetal malformations were also observed.

GoM of IC1 is the third frequent molecular finding in BWS, and accordingly it is less frequent in our prenatal cohort. In this subcohort, macrosomia and polyhydramnios are the leading features in ultrasound [16, 17].

In one pregnancy, nephromegaly, bell‐shaped thorax, and microretrognathia were observed in addition to omphalocele by ultrasound. Therefore, the suspicion for both BWS and Kagami‐Ogata syndrome (KOS14) prompted to perform Multilocus MS‐MLPA.

In a subcohort of pregnancies (*n* = 136)) with normal methylation in 11p15.5, the *CDKN1C* gene was sequenced (Table 3b, c). The frequency of pathogenic variants in this gene was 5.2% (7/136) and revealed the carrier status in four mothers, confirming that more than half of the pathogenic variants are maternally inherited [18]. Considering that pathogenic variants in *CDKN1C* are commonly associated with omphalocele and that it is associated with a 50% recurrence risk when the mother is carrier, it should be considered after negative 11p15.5 methylation testing in pregnancies with omphalocele.

## 5 Conclusions and Perspectives

Prenatal testing for BWS should be considered in pregnancies with ultrasound findings or family history suggestive for BWS as it is confirmed in 6%–13% of these pregnancies [19]. A further reason for referral is reproductive history, as assisted reproduction has been suggested to be associated with an increase probability of a child with aberrant IC2 methylation [17].

Regardless, mosaicism can never be excluded, either because its low level in the analysed sample, or because the alteration may be confined to tissue other than those tested. Therefore false‐negative results are possible and molecular BWS can never be excluded. Systematic postnatal follow‐up after prenatal testing for BWS remains challenging. The absence of integrated electronic alerts and tracking infrastructure creates a reliance on manual handover between prenatal and postnatal care teams. These barriers across the care continuum result in loss of follow‐up, which in turn hinders accurate estimation of false‐positive and false‐negative rates. As a consequence, genetic testing for BWS should be performed in case of a persisting conspicuous diagnosis of BWS after birth. Furthermore it has to be considered that BWS is a clinical diagnosis and persists even in case of a negative postnatal testing result.

When assessing the eligibility of a prenatal case for BWS testing there should be knowledge that a negative 11p15 methylation test may not have detected all causes of BWS. In particular, pathogenic sequence variants in *CDKN1C* should be considered. This gene may be individually analysed or form part of a large panel analysed in prenatal whole exome/genome sequencing which may be performed before, concurrently or after MS‐MLPA of 11p15. It is also important to ensure that when analysing the results of WES/WGS, maternal inheritance from an unaffected mother is considered. In the context of negative prenatal BWS testing, further postnatal evaluation for additional clinical features of BWS is warranted and if a suspicion of BWS remains, testing a postnatal blood sample is recommended.

Several positive cases were identified by application of MS‐MPA after a comprehensive diagnostic workup (i.e., chromosome microarray analysis, NGS/WES), illustrated by the fact that at least eight of the positively tested cases from Germany were referred for BWS testing after a negative WES trio analysis. By considering the relatively low costs of MS‐MLPA in comparison to the other tests like NGS and the time‐consuming stepwise workflow an earlier or at least parallel MS‐MLPA testing should be weighed. However, laboratories performing prenatal testing for BWS should have ample experience in testing for BWS postnatally and in the processing prenatal testing.

The methodologies for prenatal genetic testing for congenital disorders underly the same dynamics as postnatal testing, it is only a question of time that methylation‐sensitive (MS) NGS becomes routinely applied in the prenatal diagnostic workup of BWS and other imprinting disorders. Several studies have already demonstrated the feasibility of assessing imprinted loci by both short read and long read technologies [7, 8, 9], and it is likely that these techniques will be implemented in invasive and non‐invasive [20] prenatal testing. The advantage of MS NGS lies in its reduced turn‐around time through the parallel analysis of DNA sequence and imprinting marks. In the case of long read sequencing, phasing of DNA and methylation data is also possible. These approaches may enable a time‐ and cost‐efficient diagnostic workup might in the future. However, several of the challenges addressed in this paper also refer to these new techniques, in particular the limited amount and variable quality of foetal DNA and the availability of control data for methylation at different times of pregnancy.

Finally, mosaicism might remain an unsolvable issue in prenatal BWS testing, as well as the communication of incidental or unexpected findings. However, our data illustrate that prenatal testing for BWS is generally robust, but that culturing might have an impact on the reliability of the results, and that the use of tissued—matched controls might decrease the rate of failures in CVS.

## Funding

TE is supported by the Deutsche Forschungsgemeinschaft (e.g. 115/13‐1, 497659591). SR is supported by Italian Ministry of Health (08C123).

## Ethics Statement

The study was approved by the ethical committee of the Medical Faculty of the RWTH Aachen (EK303‐18).

## Consent

The patients consented that their data is included in the study.

## Conflicts of Interest

The authors declare no conflicts of interest.

## Data Availability

The data that support the findings of this study are available from the corresponding author upon reasonable request.
